# Scarlet Fever and Measles in Dogs and Cats

**Published:** 1885-01

**Authors:** John C. Peters

**Affiliations:** New York


### Art. III.—SCARLET FEVER AND MEASLES IN DOGS AND CATS.

BY JOHN C. PETERS, M.D., NEW YORK.

Copeland, Ziemssen, and many other writers allude to scarlet fever in dogs, but in such a perfunctory way that nobody pays much attention to it. Some months ago I attended a case of scarlet fever in a young lady who had a pet pug dog. It so happened that I had seen this dog several times when well; finally it sickened and seemed to have what is called "distemper," to which all young dogs are subject, like children to scarlet fever and measles. Of course I paid little or no attention to it, but recollect that it appeared to have a feverish cold with sore eyes and throat, refused its food, and rapidly fell away in condition. It was sent to a dog doctor, and soon after its young mistress had a decided outbreak of scarlet fever, about which there could not be the slightest doubt. Her eyes were affected more than is usual in scarlet fever, and the mother repeatedly called my attention to the similarity of some of the symptoms of the dog-distemper to those of her daughter. I warded off these gentle attempts to establish a connection between the disorder of the dog and that of its young mistress. Many months after, when I became interested in the possible occurrence of scarlet fever in horses, the mother again stated her belief, which she had never abandoned, that the dog had had the same disease as her daughter, and that the canine disorder, as I well knew, had the precedence in point of time. Subsequently I learned that the dog had been taken along several times by a waiter man when inquiries were made about another young lady sick with scarlet fever.

This case standing alone would, of course, form a very slender basis for anything else than to suggest that sick dogs should be examined more carefully by doctors in the future. Soon after I heard that Dr. Alexander Hadden knew something about scarlet fever in cats, and, after several conversations with him, persuaded him to write out his opinions, as follows:

"*Dear Doctor:* About fifteen years ago, my attention was called to scarlatina in animals by an article published by the well-known Dr. Snow of London, in a medical journal which I have lost, and the name of which I have forgotten. The purport of his paper was that cats were often carriers of scarlet fever to families; that they were subject to this disease, and often communicated it to their kind and also to human beings.

He also stated that they died of this malady, and then presented the common post-mortem appearances of unmistakable scarlet fever, especially in the skin and kidneys. His statements were so positive that I bore them in mind, and, when other well-known sources of the contagion were not present in the cases I met with in practice, I inquired about the condition of these common house pets. The first cases which fastened my attention to this point still more were three children in one family all attacked with scarlet fever about the same time. They had not been exposed to scarlet fever in any known way, for they had not been out of the house for several weeks, as they were snow-bound in the country and the weather was very cold. There was no scarlet fever in the neighborhood, and few or no visitors had come to the house. I asked if there were any sick cats in the family, and was told that the house cat had been ailing for a week with sore throat and eyes. The animal was sought for but could not be found, although it had been seen lying beside the stove in the morning. It never returned, and therefore I was left with incomplete evidence. The next case which presented a like suspicious history, was in a boy aged ten years, who, about four days before I was called, had been caring for a sick kitten which he had found in the street and which died on the following day. The lad had not been exposed to the contagion from any human being as far as could be ascertained; there was none in the neighborhood where he lived. His attack was well marked and very virulent, so that he died on the third day. In the following week his little sister was taken with the same disease, scarlet fever, but had it less severely and recovered. In this case, also, I was deprived of the opportunity of obtaining positive evidence for or against the suspected origin of the contagion.

"On several occasions I tried to produce scarlet fever in kittens by exposing them to the disease, but obtained no absolutely satisfactory results. The plan I pursued was to place kittens about six weeks old in bed with children suffering with scarlet fever, and allow them to remain there parts of several days. Some of these animals became sick with catarrhal affections of the eyes and throat, but none exhibited the rash upon the skin, or characteristic glandular inflammation of the neck, or marked renal conditions."

I also induced Dr. Erskine S. Bates to write out his experience:

"Dear Doctor: In looking over my case-book, I find the following notes, which may be of interest to you and others.

They are necessarily meagre, as the subject did not at that time possess the interest it will now: On March 6, 1877, I was called to attend Maggie W., aged seven. Her mother stated that the child had been very sick all night, and she was afraid she had caught some disease from a strange kitten which she had found in the hall some days previous. The kitten was sick when found, and the child had been nursing it ever since. I did not attach much importance to the mother's fears, but found that my little patient had scarlatina anginosa, and so informed the mother. The next day the mother again referred to the case of the sick kitten and told me that the family cat was now also sick, very much like the kitten, and asked me to examine both animals. I did so very skeptically, and found both cats with high fever, scarlatinous sore throats in different stages, and some discharge from the nose; they were both very sick. As the child was quite ill, little attention was given to the felines beyond watching them casually from day to day. The family cat died on the sixth day; the kitten recovered, as did the little girl. Other cases of scarlet fever subsequently developed in the same house, but I am not sure whether any other of the felidæ contracted the disease.

"Two years after, I was called to see a young man, aged twenty-eight, who had recently married and set up housekeeping in a tenement building on Avenue A. His family consisted of his wife and a pet terrier dog. The house had a bad reputation on the score of healthfulness; family after family would move in, contract some form of scarlatina, lose one or more children, and then move out. The newly-made family man had been domiciled there scarcely a week when he was attacked with scarlatina maligna, and died in forty-eight hours. The pet dog persisted in lying on the bed, as near the head of his master as he could get, all through his owner's illness; and, on the same day that his master died, was taken with convulsions, sore throat, rapid swelling of the glands of the neck, high fever, suppression of urine, etc., death closing the scene thirty-six hours after the convulsions which ushered in the attack. The train of symptoms manifested by the animal was so similar to that of his master that the mourning friends commented on the matter. I had no doubt then, as I have none now, that the dog died of scarlatina maligna. Subsequently the house was thoroughly disinfected and renovated, and I am not aware that any peculiar fatality has since attended its occupants."

Even if the animals are not sick, they may convey the poison in their fur, as was suggested by Dr. Snow, whose opinions as the originator of the whole doctrine of water-contamination in the conveyance of cholera, typhoid fever, and other diseases, are entitled to the highest respect. Dr. Janeway has reported a case in which diphtheria was conveyed from New York to Florida in the fur of a toy rabbit. Dr. Williams, President of the Royal College of Veterinary Surgeons, and Principal and Professor of Veterinary Medicine at the Veterinary College, Edinburgh, says, p. 253 of his "Principles and Practice of Veterinary Medicine," "I can compare dog-distemper to no human disease except measles, and the points of analogy are very great. In both diseases catarrhal symptoms are manifested; they are infectious; they generally occur but once in a life-time; they chiefly affect the young; in almost all cases of distemper there is some *cutaneous eruption or rash* and desquamation of the cuticle; catarrhal ophthalmia, bronchial and pulmonary inflammation, and dysentry are complications of both diseases," etc.

